# A Rare Case of Right Subclavian Artery Aneurysm Caused by Intimomedial Mucoid Degeneration at the Vertebral Artery Origin

**DOI:** 10.3400/avd.cr.26-00080

**Published:** 2026-09-18

**Authors:** Hideyuki Harada, Koji Kagawa, Tomoyuki Yokoyama, Daita Kobayashi, Takamitsu Tatsukawa, Nobuyuki Akasaka

**Affiliations:** Department of Cardiovascular Surgery, Kushiro Kojinkai Memorial Hospital, Kushiro, Hokkaido, Japan

**Keywords:** subclavian artery aneurysm, intimomedial mucoid degeneration

## Abstract

To our knowledge, this is the first reported case in Japan of a right subclavian artery aneurysm caused by intimomedial mucoid degeneration at the vertebral artery origin, successfully treated by surgical resection, with intraoperative stump pressure and cerebral oxygenation monitoring enabling safe vertebral artery ligation without reconstruction.

## Introduction

Subclavian artery aneurysms are rare conditions that can lead to life- or limb-threatening complications. Their surgical management requires careful consideration of access routes, cerebral blood flow protection, and appropriate techniques, which vary depending on the aneurysm’s location.^1–3)^ Intimomedial mucoid degeneration (IMD) is an uncommon vascular disorder characterized by mucinous deposition in the intimal and medial layers, leading to aneurysmal degeneration of the vessel wall—often observed in young patients.^4–6)^ Here, we report a case of a right subclavian artery aneurysm caused by IMD at the origin of the vertebral artery (VA) that was successfully managed with surgical intervention.

## Case Report

**Fig. 1:**
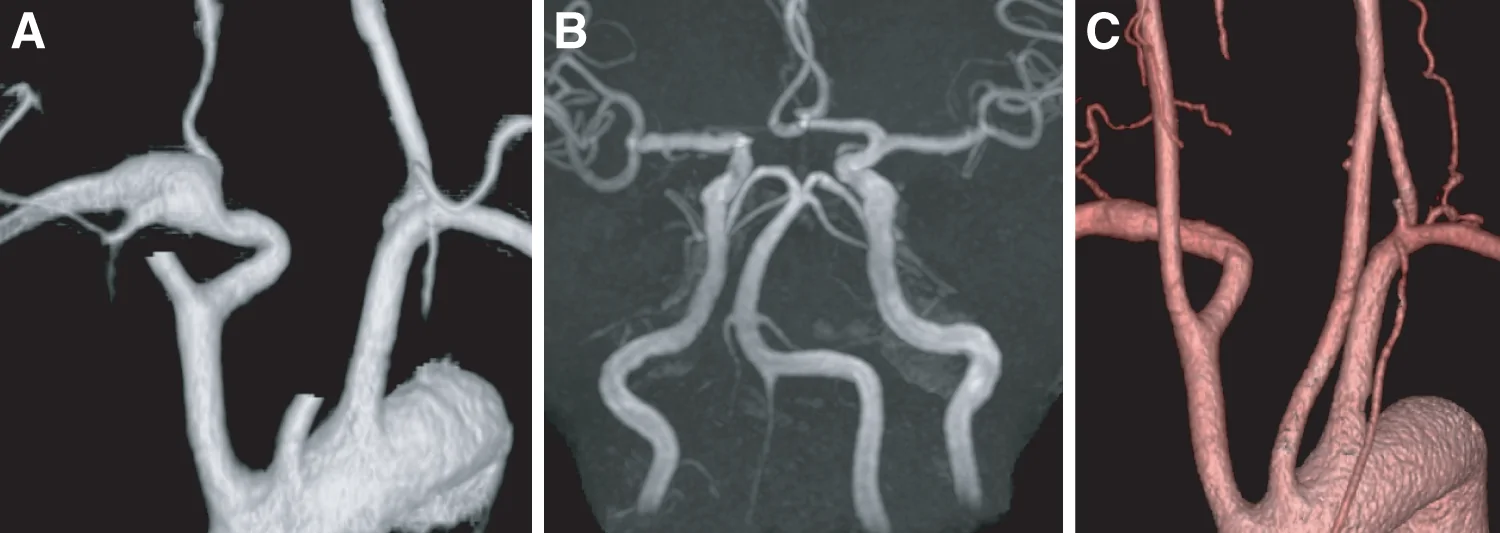
(**A**) Preoperative MRA reveals a right subclavian artery aneurysm at the vertebral artery origin. (**B**) Preoperative MRA reveals a dominant left vertebral artery. (**C**) Postoperative CTA shows good patency of the reconstruction. MRA, magnetic resonance angiography; CTA, computed tomography angiography

A 74-year-old man was referred to our hospital after a screening carotid ultrasound incidentally revealed a right subclavian artery aneurysm. The patient’s past medical history was significant for Parkinson’s disease, and there was no history of trauma. The patient was hemodynamically stable, and laboratory findings were unremarkable. Magnetic resonance angiography (MRA) demonstrated a right subclavian artery aneurysm measuring 28.2 × 17.9 × 21 mm at the VA branch; 4 years earlier, the maximum diameter had been 13.4 mm (**Fig. 1A**). MRA showed a dominant left VA (**Fig. 1B**). Considering the risk of rupture and embolism, surgical intervention was warranted, and aneurysm excision with reconstruction was planned.

**Fig. 2:**
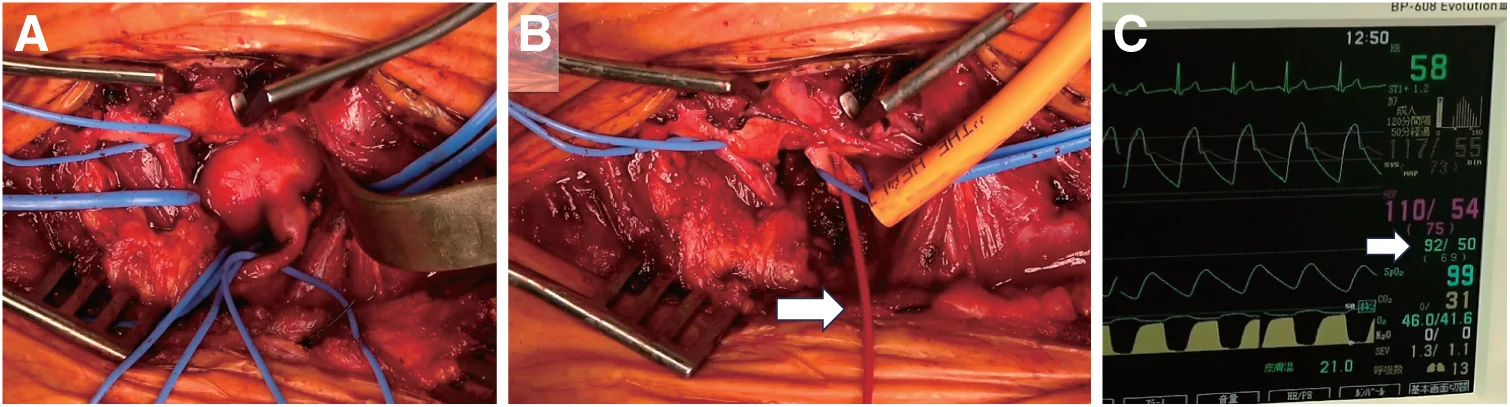
Operative findings. (**A**) The subclavian artery proximal and distal to the aneurysm, as well as the right VA, were encircled with vessel loops. (**B**) The pressure at the right VA stump was measured using a 4-F Fogarty catheter (arrow). (**C**) With a systemic blood pressure of 110 mmHg, the right VA stump pressure was 92 mmHg (arrow). VA, vertebral artery

Under general anesthesia, the patient was positioned supine with the neck rotated to the left. The upper left thigh was prepped for potential saphenous vein graft (SVG) harvesting. Intraoperative ultrasonography was used to confirm the aneurysm’s location. A 10-cm skin incision was made parallel to and 1 cm above the right clavicle. The subcutaneous tissue and platysma were dissected with electrocautery to expose the sternal head of the sternocleidomastoid muscle. This muscle was then divided parallel to the clavicle using the coagulation mode to expose the scalene fat pad. The phrenic nerve was carefully dissected free from the anterior scalene muscle and retracted using silicone tape. A partial incision of the anterior scalene muscle near its attachment to the first rib provided exposure of the right subclavian artery aneurysm. Branches of the subclavian artery—including the internal thoracic artery—were ligated, and careful dissection of the aneurysm was performed (**Fig. 2A**). The proximal and distal subclavian artery segments, along with the right VA, were encircled with vessel loops. After administering 50 mg of heparin, the subclavian artery and right VA were clamped proximally and distally to the aneurysm. The aneurysm was excised, and the proximal and distal ends of the subclavian artery were trimmed. The excised aneurysm wall was submitted for pathological examination. An end-to-end arterial anastomosis was performed using a continuous 5-0 Prolene suture. After de-airing, the clamps were released, and blood flow to the right upper limb was restored.

**Fig. 3:**
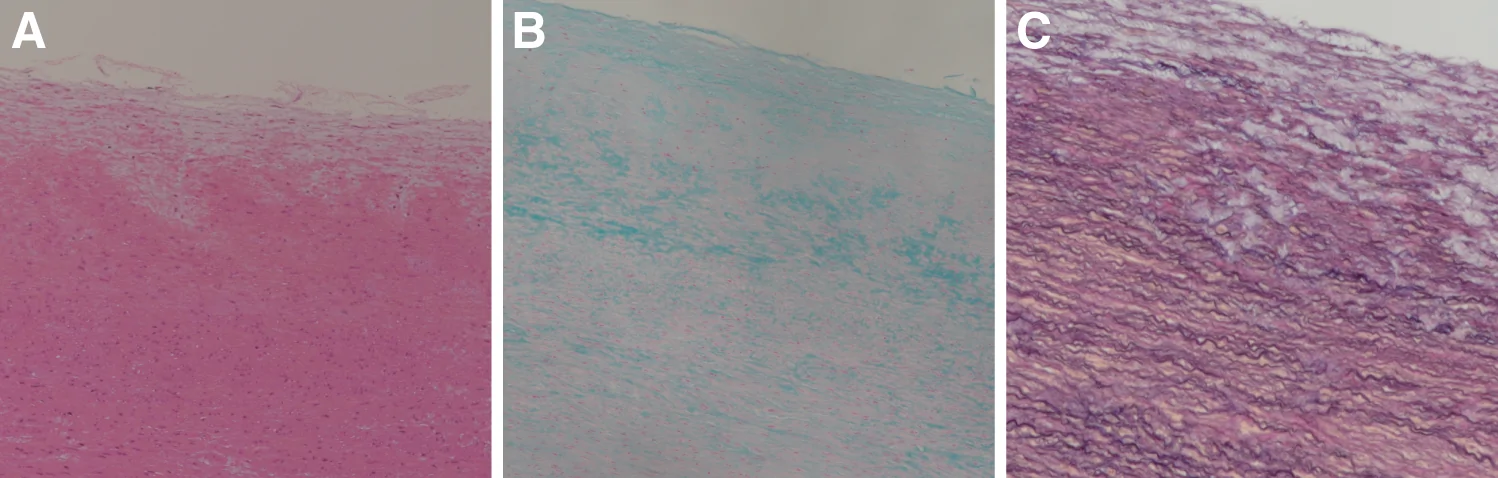
Histopathologic evaluation of the aneurysm wall. (**A**) Hematoxylin and eosin stain (×50 magnification) showing myxoid change in the intima and media. (**B**) Alcian blue stain (×50 magnification) demonstrating mucoid deposition in the intima and media. (**C**) Elastic van Gieson stain (×100 magnification) demonstrating fragmentation and aggregation of the elastic fibers.

As the left VA was dominant, the pressure at the right VA stump was measured (**Fig. 2B** and **2C**). With a systemic blood pressure of 110 mmHg, the right VA stump pressure was 92 mmHg (measured using a 4-F Fogarty catheter). No significant changes were observed in bilateral in vivo optical spectroscopy (INVOS) readings during right VA occlusion; therefore, reconstruction of the right VA was deemed unnecessary, and it was ligated with a 4-0 Prolene suture reinforced with felt. The surgical field was irrigated with saline, hemostasis was confirmed, and the muscle and fascia were closed with 2-0 Vicryl sutures. The subcutaneous tissue was closed, and the skin was approximated with 4-0 PDS sutures. Histopathologic evaluation of the resected aneurysm revealed mucoid deposition in the intima and media, confirming the diagnosis of IMD (**Fig. 3**).

The postoperative course was uneventful. The patient was extubated in the operating room and transferred to the intensive care unit (ICU). He was discharged from the ICU the following day. Postoperative CTA demonstrated no abnormalities (**Fig. 1C**), and adequate blood flow to the basilar artery was maintained via the contralateral VA. The surgical wound healed well, and the patient was discharged on postoperative day 8. At 3 years of follow-up, the patient remains healthy and maintains a normal daily life.

## Discussion

The subclavian artery is anatomically divided into proximal, middle, and distal segments. The proximal segment extends from its origin to the medial border of the anterior scalene muscle, where the VA arises from the superior surface, the internal thoracic artery from the inferior surface, and the thyrocervical trunk from the anterior surface. The middle segment lies between the medial and lateral borders of the anterior scalene muscle, while the distal segment extends from the lateral border of the anterior scalene muscle to the lateral border of the first rib.

Aneurysms in the subclavian artery can result from various etiologies. Atherosclerosis is the most common cause in the proximal segment, collagen disorders in the middle segment, and thoracic outlet syndrome in the distal segment. Trauma remains a frequent cause across all segments due to the increasing use of percutaneous catheterizations. Despite these risk factors, subclavian artery aneurysms remain exceedingly rare.^1–3)^

IMD is a relatively rare but clinically significant pathological condition that involves the deposition of mucoid substances in both the intima and media of the arterial wall, leading to progressive weakening of the vessel structure and subsequent aneurysm formation.^4–6)^ In contrast to atherosclerotic aneurysms, which are typically characterized by lipid accumulation, intimal thickening, and inflammation, IMD presents as a noninflammatory degenerative process, resulting in cystic degeneration, disruption of elastic fibers, and loss of vascular integrity.^7,8)^ The exact pathogenesis of IMD remains unclear.^9,10)^

Histopathologically, IMD is distinguished by mucoid accumulation within the intima and media, fragmentation of elastic fibers, and the absence of significant inflammatory infiltrates.^7,8)^ In the present case, additional Alcian blue staining demonstrated mucopolysaccharide deposition within the arterial wall, while the newly prepared elastic van Gieson (EVG)-stained section demonstrated disruption and fragmentation of elastic fibers (**Fig. 3**). These findings provided additional histopathological support for the diagnosis of IMD. This differs from cystic medial necrosis (CMN), seen in conditions like Marfan syndrome, which primarily affects the media with focal necrosis and elastic fiber disruption.^4,6)^ In our patient, there were no characteristic physical features or family history suggestive of Marfan syndrome.

While IMD has been widely documented in the ascending aorta and other large elastic arteries, its involvement in the subclavian artery is exceedingly rare, with very few reported cases in the literature.^1,5)^ In our case, the aneurysm was located at the origin of the right VA, a region subjected to significant hemodynamic stress due to the branching flow dynamics of the VA. This suggests that IMD may have a predilection for areas of high mechanical stress, leading to localized vascular degeneration. Given the rarity of IMD-related subclavian aneurysms, there is no standardized treatment approach. However, surgical excision remains the preferred strategy, as endovascular techniques, such as stent-graft placement, carry a high risk of endoleak, branch vessel occlusion, and continued aneurysmal degeneration due to the fragile nature of IMD-affected vessels.^2,3)^

IMD-related aneurysms pose unique challenges in both diagnosis and surgical management. In the present case, the differential diagnoses included inflammatory arterial disease, IgG4-related disease, connective tissue disorders, and segmental arterial mediolysis. The absence of significant inflammatory infiltration, the absence of characteristic physical features or family history of Marfan syndrome, and a normal serum IgG4 level (21) argued against these alternative diagnoses. Furthermore, the histopathological findings did not demonstrate the characteristic medial lysis or dissection associated with segmental arterial mediolysis. Although IMD has predominantly been reported in younger patients, the largest series by Abdool-Carrim et al. included patients ranging from 20 to 71 years of age, with a mean age of 52 years.^5)^ Our patient was 74 years old, making this case notable for his advanced age. Nevertheless, the characteristic histopathological findings, including mucopolysaccharide deposition confirmed by Alcian blue staining and disruption of elastic fibers on EVG staining, supported the diagnosis of IMD.

In our case, a comprehensive preoperative evaluation using head and neck MRA, intraoperative stump pressure measurement, and INVOS monitoring played a crucial role in guiding surgical decision-making. The decision not to reconstruct the right VA was supported by stump pressure measurement and intraoperative cerebral oxygenation monitoring, which confirmed sufficient collateral circulation from the left VA.

## Conclusion

We report a case of a right subclavian artery aneurysm caused by IMD at the VA origin. Preoperative head and neck MRA, intraoperative stump pressure measurement, and INVOS monitoring were critical in guiding successful surgical management. To our knowledge, this may be the first documented case of IMD affecting the subclavian artery in Japan, underscoring the importance of comprehensive preoperative evaluation and careful intraoperative monitoring.
